# Optical coherence tomography systems for evaluation of marginal and internal fit of ceramic reconstructions

**DOI:** 10.1080/26415275.2022.2122468

**Published:** 2022-09-29

**Authors:** Hiba Al-Imam, Ana R. Benetti, Pete Tomlins, Klaus Gotfredsen

**Affiliations:** a Department of Odontology, Section of Oral Rehabilitation, Faculty of Health and Medical Sciences, University of Copenhagen, Copenhagen, Denmark; b Department of Odontology, Section of Dental Materials, Faculty of Health and Medical Sciences, University of Copenhagen, Copenhagen, Denmark; c Caristo Diagnostics Ltd. Formerly Affiliated to Centre for Oral Bioengineering, Barts and The London School of Medicine and Dentistry, Queen Mary University of London, London, United Kingdom

**Keywords:** Optical coherence tomography, lithium disilicate, zirconia

## Abstract

**Purpose:**

To evaluate the marginal and internal fit of lithium disilicate and zirconia crowns using two optical coherence tomography (OCT) systems in order to estimate inter-system variations.

**Materials and methods:**

Ten lithium disilicate and 10 cubic stabilized zirconia crowns were placed on prepared artificial teeth without cement. Marginal discrepancy and internal cement gap of the crowns were assessed on images obtained using a swept source OCT (SS-OCT) and a spectral domain OCT (SD-OCT). Medians and interquartile ranges were calculated for both materials and OCT systems. Thereafter, Wilcoxon signed rank test was carried out.

**Results:**

No significant difference was found between the two OCT systems for absolute marginal discrepancy of either lithium disilicate (SS-OCT: 182 µm, SD-OCT: 214 µm; *p* = .922) or zirconia crowns (SS-OCT: 116 µm, SD-OCT: 121 µm; *p* = .232). Regarding internal cement gap, no significant difference was found between the two OCT systems for lithium disilicate crowns (SS-OCT: 128 µm, SD-OCT: 128 µm; *p* = .064). However, for zirconia crowns the SD-OCT showed significantly higher (*p* = .027) internal cement gap (92 µm) than the SS-OCT (68 µm). Moreover, it was not possible to assess the internal fit of zirconia crowns in 47% and 34% of the sites using SD-OCT and SS-OCT, respectively.

**Conclusions:**

No significant difference was noted in the ability of SS-OCT and SD-OCT to assess the marginal and internal fit of lithium disilicate crowns, nor the marginal fit of zirconia crowns. On the contrary, drawbacks regarding the assessment of internal fit of zirconia crowns using both OCT systems were observed.

## Introduction

Optical coherence tomography (OCT) is an optical technology that allows non-invasive, in-depth imaging of dental hard tissues and dental materials [1]. OCT uses near infrared light with wavelengths between 800 and 1500 nm to produce two-dimensional images of a desired sample with micrometre resolution [1–3].

In dentistry, OCT was mainly used in diagnostics of dental hard tissues and cariology [1]. However, the use of OCT has increased markedly in the different fields of dentistry including dental materials and prosthodontics [1]. In prosthodontics, OCT has been used as a diagnostic tool to assess ceramic-enamel interfacial debonding [1,4], defects of reconstructions [5] and marginal and/or internal adaptation of reconstructions produced in acrylic [6] or different dental ceramics [4,7–10]. Studies demonstrated OCT to be a reliable and valid tool for evaluating marginal and internal fit of such dental reconstructions [4,6–9]. Nevertheless, since only materials that can transmit light are suitable to be scanned with OCT, the optical properties of the reconstruction materials shall be considered. An assessment of different dental materials used in prosthodontics will demonstrate the full potential and/or limitations of OCT as a new diagnostic approach to evaluate the fit of reconstructions. In this context, it would be of interest to further assess lithium disilicate and high translucent zirconia, materials that are largely used in the current dental practice. Lithium disilicate is known for its high translucency (refractive index; *n* = 1.55) making it suitable to be assessed with OCT, as shown in previous studies [7,8]. More recently, the potential use of OCT in the assessment of thin zirconia reconstructions was demonstrated [9,10] granted the development in zirconia’s optical properties (refractive index; *n* = 2.177–2.088) [11,12].

Nonetheless, different types of OCT systems have been used to assess the fit of dental reconstructions since 2018 [6–10]. OCT is based on light interference between signals from a sample and a reference mirror [13–15]. Depending on the OCT system, the near infrared light can either be swept source (SS-OCT) or broadband. In the SS-OCT, a tunable laser is used to sweep the wavelengths. Broadband source is applied in the spectral domain OCT (SD-OCT) system and emits a broad range of wavelengths. In both systems, a beam splitter is used to split the light beam in two, propagating to the reference mirror and to the sample. Subsequently, the light backscattered from within the sample and from the reference mirror is coupled through the beam splitter/coupler [13–15]. In the specific SD-OCT system employed in this study, the interference fringes from the reference mirror and sample are detected using a diffraction grating and a single-line photodetector. However, other configurations may be applied in SD-OCT systems [13,15]. For the SS-OCT system, the light is detected using a single-element balanced photodetector. These interference fringes provide a single A-scan, which is a single scanned line in depth from within a sample. Raster scanning across the sample will result in propagation of A-scans, giving rise to a 2D in depth image – known as a B-scan.

Whilst some studies used a SD-OCT with central wavelengths of 840, 930, or 1310 nm to respectively assess the marginal fit of lithium disilicate crowns [8] and inlays [7] or leucite-containing ceramic veneers [4], others used a SS-OCT with central wavelengths of 1300 or 1310 nm to independently assess the fit of thin high translucent zirconia structures (0.5 mm-thickness) [9,10] or temporary bridges [6]. As the range of values for marginal and internal fit may differ depending on the measuring technique [10], and given the fact that the OCT systems differ in respect to light source and configuration [13–15], it is of high value to assess whether practical aspects of implementation will lead to differences between the two OCT methods. For example, the OCT systems can differ in spectral shape and bandwidth, which affect the axial resolution. Proper validation and calibration [6,16] of the OCT systems – either by using point spread function (PSF) phantoms for OCT imaging or by imaging an object with known dimensions – are therefore paramount to ensure trustworthy results.

Moreover, the maximum imaging depth is dependent upon the light source centre wavelength and the scattering properties of the sample under investigation [13–15]. At wavelengths below 1000 nm, the light penetration depth becomes more dependent on the scattering properties of the investigated sample [14]. As higher wavelengths can reduce scattering and increase depth of penetration, their use to assess fit of ceramic reconstructions is worth investigating further. Additionally, the performance of OCT systems in assessing the fit of ceramic reconstructions is not yet fully explored.

Thus, the aim of this study was to evaluate the marginal and internal fit of lithium disilicate and high translucent zirconia crowns using two different OCT systems in order to estimate inter-device variations. The null hypothesis for this study was that no difference exists in the median absolute marginal discrepancy and internal cement gap of the ceramic crowns measured with the two OCT systems.

## Material and methods

Ten plastic injection moulded Frasaco molar teeth (no. 16) with 1 mm chamfer preparation were scanned with an intraoral scanner (Trios, 3Shape A/S, Copenhagen, Denmark). Ten lithium disilicate crowns (e.max CAD, IvoclarVivadent, Schaan, Liechtenstein) and 10 cubic stabilized zirconia crowns (Ceramill Zolid FX Multilayer, Amann Girrbach AG, Koblach, Austria) were produced. The sample size was calculated [17] for a power of 0.9, an error probability of 0.05, a clinically relevant difference of 40 μm and a standard deviation (SD) of 25 μm, the latter found in a previous study [6].

The crowns were individually designed using the Dental System CAD software (3Shape A/S, Copenhagen, Denmark). The lithium disilicate crowns were milled using a 4-axis milling machine (Roland DWX-4W, Roland DGA Corporation, Irvine, CA, USA) and the zirconia crowns were milled using a 5-axis milling machine (Sirona MC XL, Sirona Dental Systems GmbH, Bensheim, Germany). The cement gap from the margin line and up to 800 µm into the preparation was set at 20 µm. The cement gap on the rest of the preparation was set at 60 µm. The smoothing distance, which is the distance between a cement gap of 20 and 60 µm, was set to 200 µm. Drill radius used for drill compensation was set to 550 µm. These settings were equal for both milling machines. To avoid chipping during milling, the lithium disilicate crowns were milled with a horizontal width at the crown margin (margin line offset) of 200 µm whilst for the zirconia crowns this was set at 150 µm.

Two validated OCT systems (SS-OCT and SD-OCT) were used to assess the absolute marginal discrepancy and the air space between the ceramic crowns and the preparations, i.e. the internal cement gap. The employed SS-OCT is a commercial OCT (Santec IVS-300, Komaki, Japan) with a center wavelength of 1310 ± 30 nm and a depth of field of 1.6 mm in air. The SS-OCT was prior to this study validated on an object of known dimensions and highly correlates to the replica technique in the assessment of fit of dental reconstructions [6]. The other OCT is a non-commercial SD-OCT (Queen Mary University of London, London, UK) with a center wavelength of 1325 ± 50 nm and depth of field of 1.2 mm in air. The custom made SD-OCT was thoroughly calibrated in an earlier study using PSF phantoms for OCT imaging, which are made of solid clear polyurethane containing sub-micrometre iron oxide particles [16]. Since both OCT systems show proved accuracy [6,16], i.e. trueness (closeness to reference values) and precision (good agreement to a set of results), no additional methods for comparison of results were deemed necessary.

**Figure 1.**
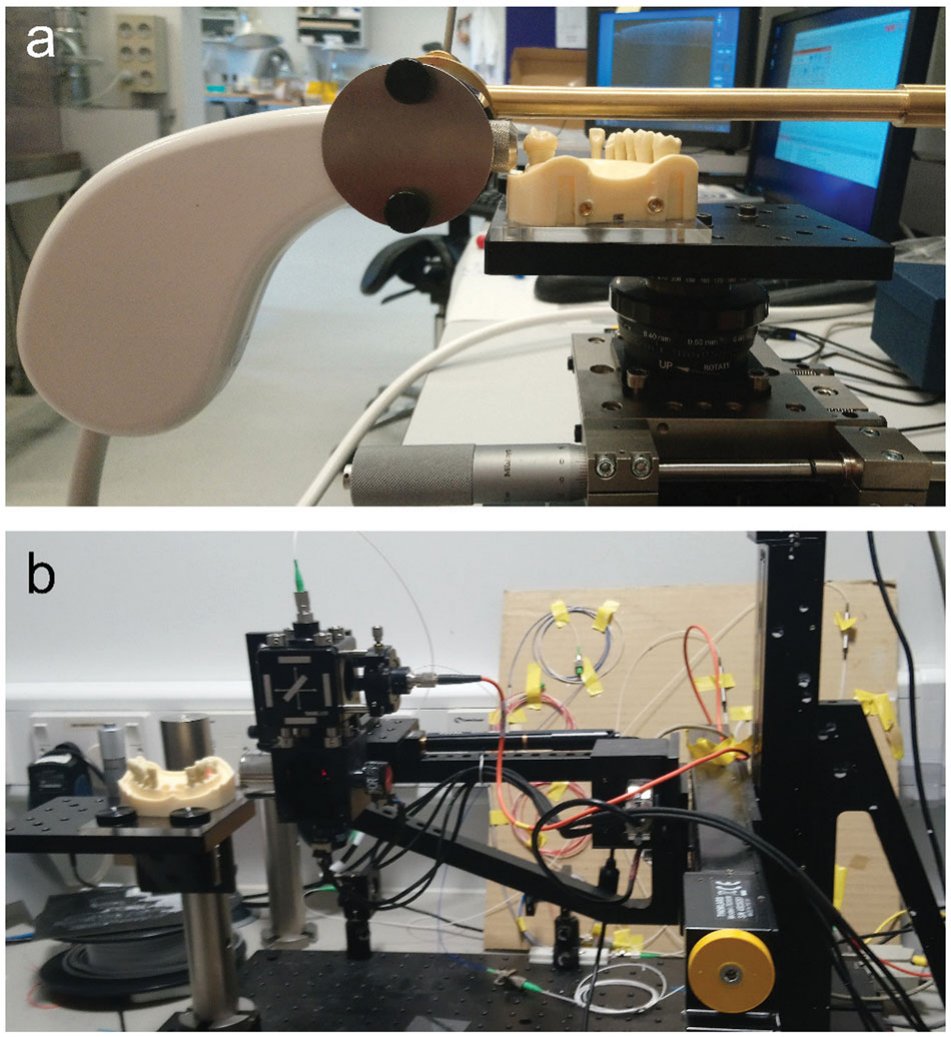
Experimental set-up using (a) the commercial SS-OCT (Santec IVS-300, Japan) and (b) the non-commercial SD-OCT (Queen Mary University of London, London, UK).

**Figure 2.**
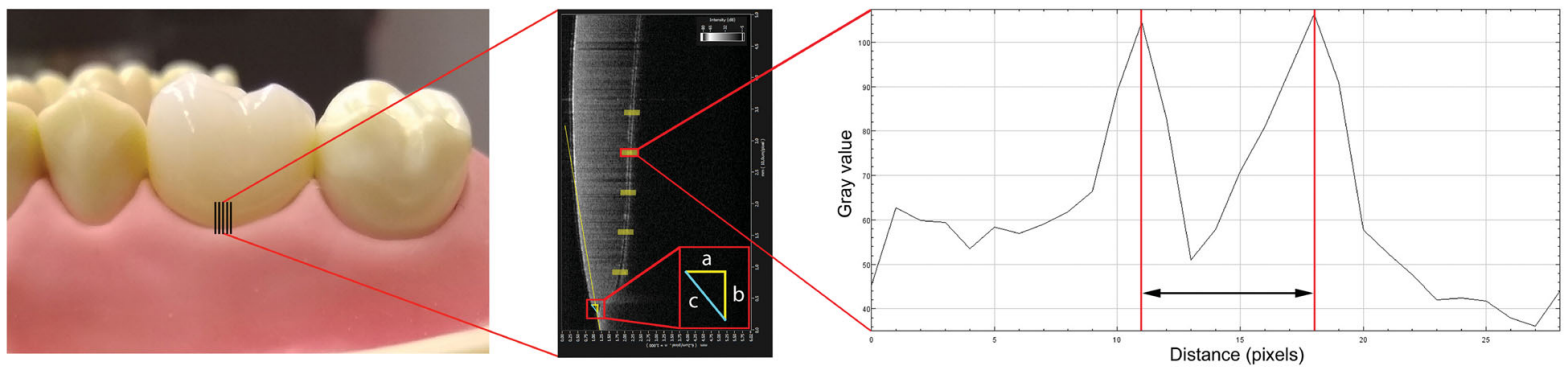
Details of measurement method: black lines cervically on the ceramic crown placed on the abutment tooth indicate the area for OCT imaging (left). B-scans (center) were obtained from five sites at the facial surface, 250 µm apart. On the B-scans, measurement sites were defined. Lines delimiting the marginal horizontal (a) and vertical (b) discrepancies were used to calculate the absolute marginal discrepancy (c), and measurement areas were delimited for assessment of internal fit. The internal cement gap was defined as the distance between two peaks on the gray value intensity plot (right).

Each prepared tooth was fixed on the phantom model (FRASACO GmbH, Tettnang, Germany). The phantom model was then securely placed on a custom made board that ensured that the model was always in the same orientation. The custom board was mounted onto a computer-controlled motorised linear translation stage (Thorlabs Inc., Newton, NJ, USA) with a repositioning accuracy of 2 µm, in order to ensure high repeatability (Figure 1(a,b)). Reference points were made on tooth 14, which were used as the start position for the motorised linear translation stage. The fixed reference points placed on tooth 14 ensured that the translation stage moved consistently so the measurements were always acquired on the same locations and within the same distance on all 10 prepared teeth no. 16 (Figure 2).

**Figure 3.**
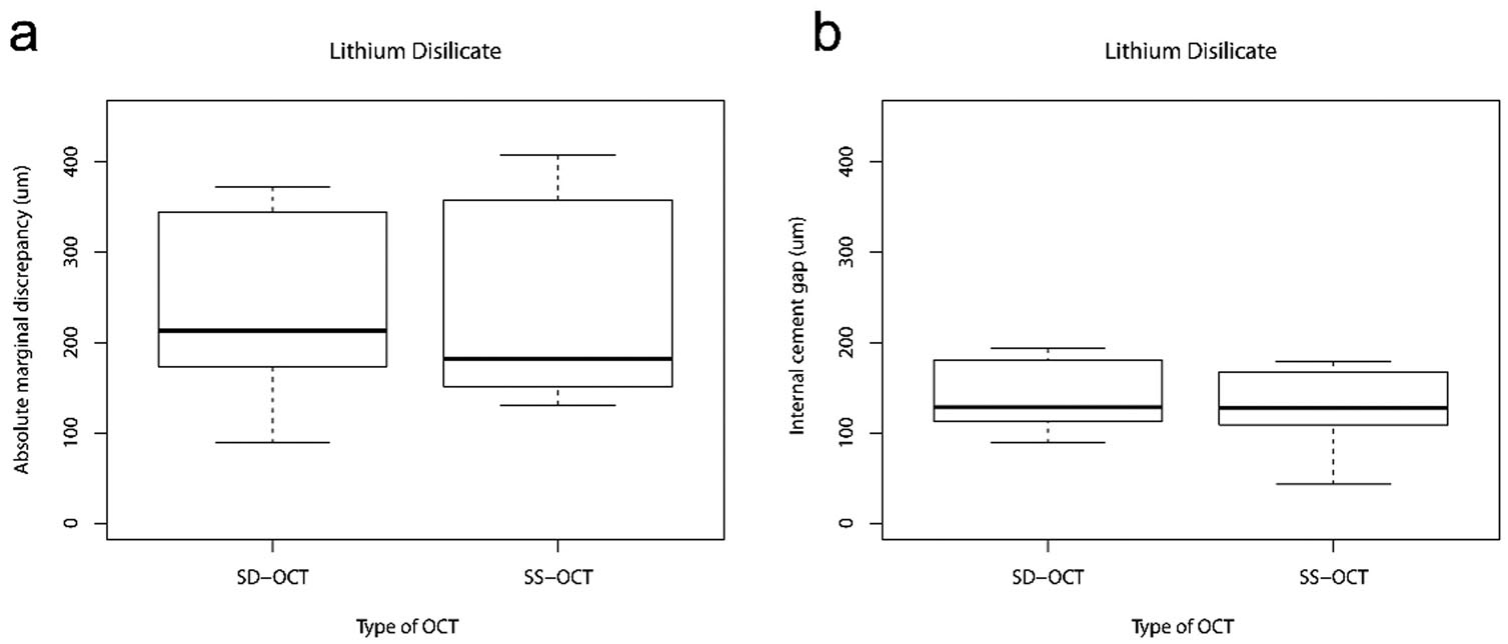
Box-plots representing (a) absolute marginal discrepancy (µm) and (b) internal cement gap (µm) of lithium disilicate crowns assessed by SD-OCT and SS-OCT.

**Figure 4.**
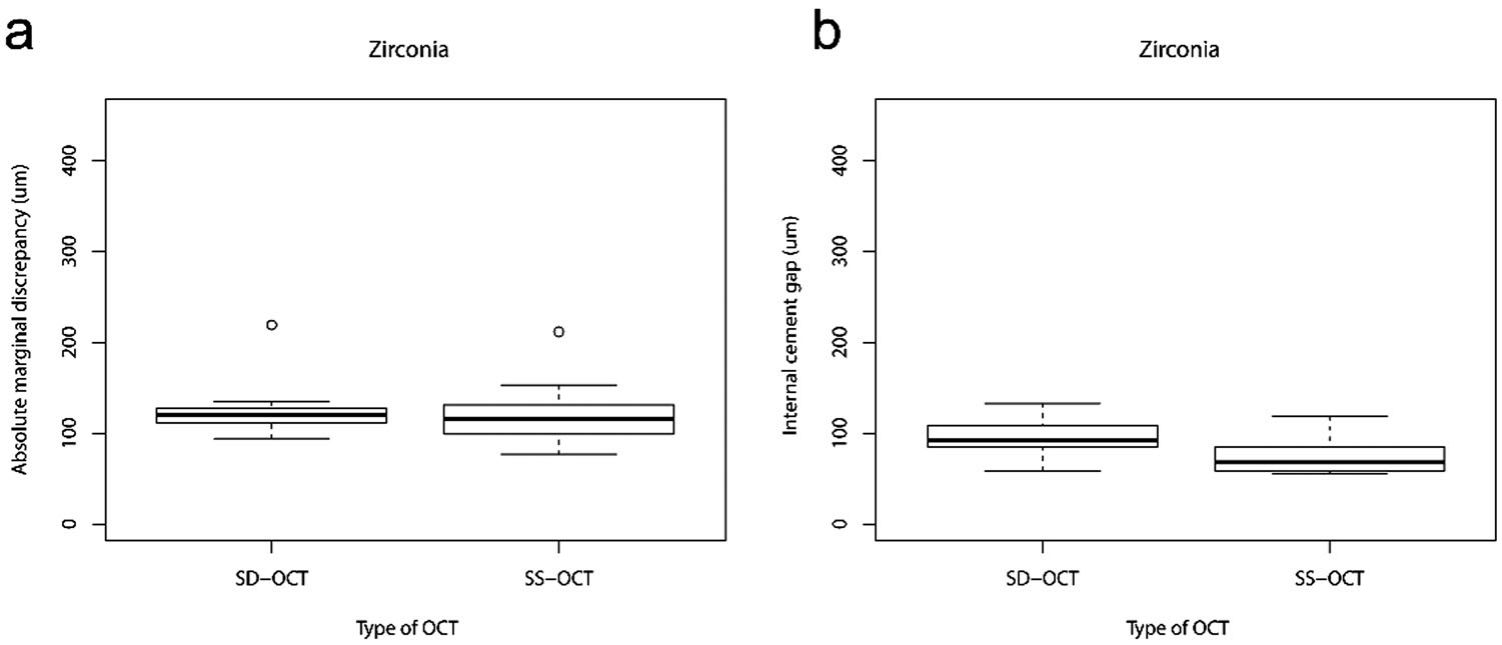
Box-plots representing (a) absolute marginal discrepancy (µm) and (b) internal cement gap (µm) of zirconia crowns assessed by SD-OCT and SS-OCT.

The ceramic crowns were placed on the prepared teeth and scanned with the two different OCT systems in turn. For each OCT system, five B-scans were acquired from the central buccal surface of each crown with a distance interval of 250 µm (Figure 2). The B-scans were then transferred to the software ImageJ (National Institutes of Health, Bethesda, MD, USA) for analysis of the marginal and internal fit.

A line tangent to the tooth surface was used to delimitate the preparation margin in the OCT images. The height of the gap (from preparation margin to crown) and the underextended or overextended margin were measured and used to calculate the absolute marginal discrepancy (i.e. the hypotenuse of the triangle, Figure 2) inspired by the method proposed by Holmes et al. [18] and applied in a previous study [6].

The internal fit was analysed by measuring the internal cement gap on the z-axis at distances of 0.5, 1, 1.5, 2 and 2.5 mm from the preparation margin and on each B-scan (Figure 2).

A grey-scale intensity value plot was then used to measure the cement gap [6]. The inner side of the crown and the outer surface of the abutment resulted in higher backscattered light, thus resulting in two peaks in the grey scale intensity value plot. Thus, the internal cement gap was measured from peak to peak in pixels (Figure 2). If one or both peaks were not visible, it was registered as a missing value in the data set. The internal cement gap was then calculated in micrometres by multiplying the number of pixels with the calibrated pixel size on the z-axis (16.8 μm/pixel for the SS-OCT and 19.9 μm/pixel for the SD-OCT) and dividing it with the refractive index of air (*n* = 1.0). For each crown, 5 OCT B-scans were obtained and for each B-scan 5 internal cement gap measurements were made, giving a total of 25 values per crown. The missing values were counted and percentages of missing internal cement gap values were calculated for each OCT system and each crown material.

Error of the measuring protocol was assessed by calculating the SD of 10 measurements made at each step of the protocol. Thus, SD was calculated for positioning the phantom model according to the reference points, which resulted in a SD of ± 34 µm for the SS-OCT and ± 7 µm for the SD-OCT. The SD calculated for seating an arbitrarily chosen crown on the phantom model was ± 11 µm. Regarding the measuring protocol performed in ImageJ, SD was calculated for finding the preparation margin from 10 tangent measurements on an arbitrarily selected B-scan, registered as SD ± 7 μm. Subsequently, the SD calculated for the assessment of grey scale intensity value plot for internal cement gap evaluation was 12 μm.

Furthermore, the error estimation in measuring the internal cement gap perpendicularly - considering the convergence angle of the prepared tooth and the curvature of the crowns - was based on the assumption that the internal cement gap would be 60 μm when measured at normal light incidence, and that the axial wall of the preparation had a convergence angle of 10°. Thus, by using the cosine function, the internal cement gap was expected to deviate by a maximum of 1 μm.

By assuming that the incident angle of light is normally distributed and the difference in angles is linearly related, the error contribution of the incident angle at 10° is estimated to be 1 µm. The square root of the sum of all squared SDs was calculated giving a total SD of ± 38 µm for the SS-OCT and ± 19 µm for the SD-OCT. Subsequently, the total standard error (SEM) of the measuring protocol was calculated as 12 μm for the SS-OCT and 6 µm for the SD-OCT.

Data analysis was performed using the statistical program R (R Core Team, Vienna, Austria). Shapiro–Wilk test was used to test for the normality of the data. As the data was not normally distributed, medians and interquartile ranges (IQR) were calculated for the absolute marginal discrepancy and the internal cement gap for all ceramic crowns. Thereafter, non-parametric Wilcoxon signed-rank test was performed to compare the data.

## Results

Analysis of the absolute marginal discrepancy and internal cement gap of monolithic lithium disilicate and zirconia crowns using the SS-OCT and the SD-OCT are shown in Figures 3 and 4. None of the ceramic crowns were underextended. The measured height of the gap (considered vertically from the preparation margin towards the crown margin) and the measured overextended margins (considered horizontally from the preparation margin towards the facial surface of the crown) are shown in Table 1. No significant difference was found between the SS-OCT and the SD-OCT in the absolute marginal discrepancies of lithium disilicate (*p* = .922) and zirconia crowns (*p* = .232). The SS-OCT detected a median absolute marginal discrepancy of 182 µm (IQR 197 µm) and the SD-OCT detected a median absolute marginal discrepancy of 214 µm (IQR 138 µm) for lithium disilicate crowns. For zirconia crowns, the SS-OCT detected a median absolute marginal discrepancy of 116 µm (IQR 29 µm) and the SD-OCT detected a median absolute marginal discrepancy of 121 µm (IQR 15 µm).

**Figure 5.**
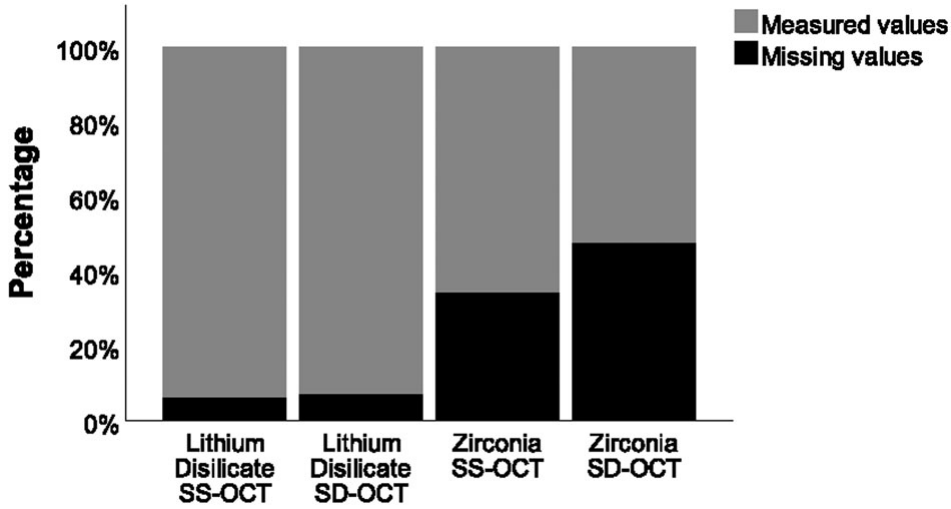
Percentage of measured and missing internal cement gap values for lithium disilicate and zirconia crowns according both OCT system.

**Table 1.** Median and interquartile ranges (IQR) of height of marginal gap (i.e. straight vertical line from preparation margin towards crown margin) and overextended margins (i.e. straight horizontal line from preparation margin towards facial surface of the crown) of lithium disilicate and zirconia crowns using Swept Source OCT (SS-OCT) and Spectral Domain OCT (SD-OCT).

	Height of gap median (IQR)	Overextended margin median (IQR)
Lithium disilicate SS-OCT	42 (95)	179 (188)
Lithium disilicate SD-OCT	64 (92)	193 (89)
Zirconia SS-OCT	NI^a^	116 (29)
Zirconia SD-OCT	NI^a^	119 (153)

The values are given in micrometer.

^a^NI: not identified with OCT.

The SS-OCT showed a median internal cement gap of 128 µm (IQR 49 µm), very close to the values obtained with the SD-OCT (median 128 µm, IQR 66 µm). No significant difference was found in internal fit between the two OCT systems (*p* = .064) for lithium disilicate crowns. Assessment of the internal cement gap of zirconia crowns showed significantly higher (*p* = .027) values (median 92 µm, IQR 21 µm) using SD-OCT than SS-OCT (median 68 µm, IQR 23 µm).

Additionally, a higher percentage of missing values (Figure 5) was registered for the SD-OCT (lithium disilicate 7% and zirconia 47%) than the SS-OCT (lithium disilicate 6% and zirconia 34%).

## Discussion

The SS-OCT and the SD-OCT identified similar marginal fit of lithium disilicate (difference of 10 µm between results obtained with the two systems) and zirconia crowns (difference of 3 µm between results obtained with the two systems). The absolute marginal discrepancy found in the lithium disilicate group exceeded the suggested clinical acceptable marginal gap of 120 µm [8]. Although the clinically acceptable marginal gap does not take the over- and under-extension (i.e. horizontal discrepancy) into consideration, our values were higher compared with two earlier studies, which found absolute marginal discrepancy of lithium disilicate crowns below 120 µm [19,20]. Since the crown margins of both ceramic materials were not adjusted after milling, this explains the high value of the overextended margins – as the predefined margin line offset influenced the absolute marginal discrepancy. Furthermore, the predefined cement gap near the preparation margin was set to 20 µm while the cement gap was set to 60 µm in the rest of the preparation. Other studies have used cement gaps ranging from 0 to 150 µm [7,8,21,22]. Miwa et al. evaluated the marginal gap of lithium disilicate crowns using different predefined cement gaps (90, 120 and 150 µm) and found that a predefined cement gap of 90 µm resulted in the largest marginal gap measured as 157 µm [21]. This confirms that the marginal fit of ceramic reconstructions is not only affected by the predefined cement gap settings but also by the accumulation of errors throughout the whole fabrication process: from data processing to the manufacture of the final crown [6,23]. In this study, the design parameters were the same for both ceramic materials; however, different milling machines were used for the two types of ceramic crowns and two different technicians produced the crowns. Due to the differences in the fabrication process, the two ceramics were not compared in our study.

The internal cement gap found for lithium disilicate crowns deviated 68 µm from the predefined cement gap (60 µm) while for zirconia the values deviated between 8 and 32 µm. The high deviation in internal cement gap in lithium disilicate reconstructions was also found in another study, which showed internal cement gap values obtained with the SD-OCT to be ∼90 µm higher than the predefined cement gap of 10 µm [7]. Miwa et al. reported internal cement gap values to deviate 90, 41 and 34 µm from the predefined cement gaps of 90, 120 and 150 µm, respectively [21]. Regarding the zirconia crowns in this study, deviation between the internal cement gap (33 µm) and the predefined cement gap of 60 µm was found to be similar to values found in an earlier study [22]. It is worth to mention that, in contrast to our study, which measured the internal cement gap without cement, other studies evaluated the internal fit of reconstructions after cementation or by using the replica technique [4,7,8,21,22]. Furthermore, in this study, the measured area was limited to the central buccal surface of the crowns and the internal fit was only assessed within 2.5 mm from the margin line.

As a significant difference was found between the SS-OCT and the SD-OCT in the internal fit of zirconia crowns, the null-hypothesis was partially rejected. A high number of missing values, i.e. immeasurable internal cement gaps, was registered for zirconia crowns using the SD-OCT. The missing internal cement gaps reflect the difficulty in obtaining information at the zirconia-tooth interface. The light penetration depth is dependent on the scattering properties of the ceramic crowns and the OCT light source [13–15]. The change of refractive index across the interface between grain boundaries, pores, impurities and crystallographic orientations increase light scattering, which in turn decrease the translucency [9,10,24,25]. Moreover, reflection and absorption of light also reduce light penetration depth [11,12]. Although these factors have been improved in cubic stabilized zirconia, light scattering and the refractive index of these materials are still higher than lithium disilicate [26], which help explain why the percentage of missing values were higher in zirconia crowns than in lithium disilicate crowns. Furthermore, the zirconia crowns were more convex on the buccal surface than the lithium disilicate crowns, which affected the angle of incident light and subsequently the internal cement gap measurement. Such important limitations were not reported earlier, and must be considered in future work and during the development of OCT systems for dental applications. Otherwise, the validity of using OCT to assess the fit of zirconia reconstructions becomes questionable.

In this study, the two calibrated OCT systems operated with similar center wavelengths and assessed the same samples. Thus, it was assumed that the potential maximum axial penetration depth and resolution to be similar. However, the depth of field was not the same for the two OCT systems. The SD-OCT is a custom-made system that was originally set-up for erosion monitoring, thus having the depth of field focused at the top of the B-scan [27]. Thus, the B-scans obtained with the SD-OCT seemed more focused on the surface of the crowns, making it easier to measure the marginal fit, and less focused around the interface between crown and tooth. In the SS-OCT, the surface of the crowns near the margin was more blurry; however the B-scans were sharper at the interface between crown and tooth. The less sharp surface observed using the SS-OCT can help explain the higher error associated with positioning the phantom model according to the reference points, compared with the SD-OCT. The reference points were sharper and easier to detect on the B-scans obtained with the SD-OCT.

Other important factors that may explain the differences in results obtained from the two OCT systems are the numerical aperture of the imaging optics and the configuration of imaging and detection optics. The numerical aperture influences the maximum imaging depth and resolution, whereas imaging and detection optics influence the pixel sizes [13]. The pixel sizes can vary non-linearly across a B-scan [16] and a thorough resolution assessment was not performed in this study. However, the axial and lateral pixel sizes were calibrated in the area where the measurements were obtained for both OCT systems.

In addition, the sensitivity of the OCT systems also influences the imaging depth and resolution [13]. The sensitivity of the two OCT systems was not investigated in this study, but SS-OCT has been reported to have a higher signal-to-noise ratio (SNR), thus greater sensitivity [2,16], than SD-OCT. This may further explain why the SS-OCT had less missing values than the SD-OCT.

All of the mentioned factors can introduce uncertainty into physical measurements and contribute to the difference in results between the two investigated OCT systems. However, not all differences in the results obtained with the SS-OCT and the SD-OCT can be solely attributed to wavelength and OCT configuration. Other factors, such as the setup, the positioning of the phantom model, the method used for image analysis, all quantified in this study, contribute to measurement uncertainty.

In summary, since OCT systems have different configurations, it is of great importance to assess factors, such as the instrument resolution, depth of field, sensitivity, pixel size and wavelength in order to find the most suitable OCT for assessing the fit of dental reconstructions. Both OCT systems generally agreed to within ∼30 µm for absolute marginal discrepancy and within ∼25 µm for internal cement gap. The clinical relevance of these differences are of minor importance and considering the contemporary methods to inspect marginal and internal fit of reconstructions in the dental clinic, OCT is able to identify much smaller discrepancies than radiography or clinical probing.

## Conclusions

The new approach of using OCT for measuring marginal and internal fit of ceramic reconstructions worked well for lithium disilicate reconstructions, where both systems showed equivalent performance. Whilst similar results were observed during the assessment of marginal fit of cubic stabilized zirconia crowns, differences between the two OCT systems and a high percentage of unmeasurable sites were noted when assessing the internal fit of zirconia with OCT.

Considering the clinical benefits provided by OCT in the assessment of dental materials and fit of reconstructions when compared to the available methods used in dental practice, the limitations observed during OCT imaging of zirconia crowns open the possibility for further research in the field.

## References

[1] Machoy M, Seeliger J, Szyszka-Sommerfeld L, et al. The use of optical coherence tomography in dental diagnostics: a state-of-the-art review. J Healthc Eng. 2017;2017:7560645. 29065642 10.1155/2017/7560645 PMC5534297

[2] Clarkson DM. An update on optical coherence tomography in dentistry. Dent Update. 2014;41(2):174–176. 24783887 10.12968/denu.2014.41.2.174

[3] Schmitt JM. Optical coherence tomography (OCT): a review. IEEE J Select Topics Quantum Electron. 1999;5(4):1205–1215. 10.1109/2944.796347 PMC4358303 25774083

[4] Haak R, Siegner J, Ziebolz D, et al. OCT evaluation of the internal adaptation of ceramic veneers depending on preparation design and ceramic thickness. Dent Mater. 2021;37(3):423–431. 33288325 10.1016/j.dental.2020.11.021

[5] Shimada Y, Sadr A, Sumi Y, et al. Application of optical coherence tomography (OCT) for diagnosis of caries, cracks, and defects of restorations. Curr Oral Health Rep. 2015;2(2):73–80. 26317064 10.1007/s40496-015-0045-z PMC4544493

[6] Al-Imam H, Michou S, Benetti AR, et al. Evaluation of marginal and internal fit of acrylic bridges using optical coherence tomography. J Oral Rehabil. 2018;03:1–8. 10.1111/joor.12746 30387868

[7] Turk AG, Sabuncu M, Ulusoy M. Evaluation of adaptation of ceramic inlays using optical coherence tomography and replica technique. Braz Oral Res. 2018;32:e005. 29412222 10.1590/1807-3107BOR-2018.vol32.0005

[8] Li W, Liu J, Zhang Z. Evaluation of marginal gap of lithium disilicate glass ceramic crowns with optical coherence tomography. J Biomed Opt. 2018;23(3):1–5. 10.1117/1.JBO.23.3.036001 29500872

[9] Lee S, Son K, Park J, et al. Non-ionized, high-resolution measurement of internal and marginal discrepancies of dental prosthesis using optical coherence tomography. IEEE Access. 2019;7:6209–6218.

[10] Son K, Lee S, Kang SH, et al. A comparison study of marginal and internal fit assessment methods for fixed dental prostheses. J Clin Med. 2019;8(6):785. 10.3390/jcm8060785 PMC6617221 31159460

[11] Zhang Y. Making yttria-stabilized tetragonal zirconia translucent. Dent Mater. 2014;30(10):1195–1203. 25193781 10.1016/j.dental.2014.08.375 PMC4167579

[12] Shahmiri R, Standard OC, Hart JN, et al. Optical properties of zirconia ceramics for esthetic dental restorations: a systematic review. J Prosthet Dent. 2018;119(1):36–46. 28927925 10.1016/j.prosdent.2017.07.009

[13] Tomlins PH, Wang RK. Theory, developments and applications of optical coherence tomography. J Phys D Appl Phys. 2005;38(15):2519–2535.

[14] Hsieh YS, Ho YC, Lee SY, et al. Dental optical coherence tomography. Sensors. 2013;13(7):8928–8949. 23857261 10.3390/s130708928 PMC3758630

[15] Podoleanu AG. Optical coherence tomography. J Microsc. 2012;247(3):209–219. 22708800 10.1111/j.1365-2818.2012.03619.x PMC3563006

[16] Fouad A, Pfefer TJ, Chen CW, et al. Variations in optical coherence tomography resolution and uniformity: a multi-system performance comparison. Biomed Opt Express. 2014;5(7):2066–2081. 25071949 10.1364/BOE.5.002066 PMC4102349

[17] Kadam P, Bhalerao S. Sample size calculation. Int J Ayurveda Res. 2010;1(1):55–57. 20532100 10.4103/0974-7788.59946 PMC2876926

[18] Holmes JR, Bayne SC, Holland GA, et al. Considerations in measurement of marginal fit. J Prosthet Dent. 1989;62(4):405–8408. 2685240 10.1016/0022-3913(89)90170-4

[19] Dolev E, Bitterman Y, Meirowitz A. Comparison of marginal fit between CAD-CAM and hot-press lithium disilicate crowns. J Prosthet Dent. 2019;121(1):124–128. 29961628 10.1016/j.prosdent.2018.03.035

[20] Min-Kyung J, Park JH, Sang-Won P, et al. Evaluation of marginal fit of 2 CAD-CAM anatomic contour zirconia crown systems and lithium disilicate glass-ceramic crown. J Adv Prosthodont. 2015;7(4):271–277. 26330973 10.4047/jap.2015.7.4.271 PMC4551782

[21] Miwa A, Kori H, Tsukiyama Y, et al. Fit of e.max crowns fabricated using conventional and CAD/CAM technology: a comparative study. Int J Prosthodont. 2016;29(6):602–607. 27824983 10.11607/ijp.4865

[22] Berrendero S, Salido MP, Valverde A, et al. Influence of conventional and digital intraoral impressions on the fit of CAD/CAM-fabricated all-ceramic crowns. Clin Oral Investig. 2016;20(9):2403–2410. 10.1007/s00784-016-1714-6 26800669

[23] l-Imam H, Gram M, Benetti AR, et al. Accuracy of stereolithography additive casts used in a digital workflow. J Prosthet Dent. 2018;119(4):580–585. 28781073 10.1016/j.prosdent.2017.05.020

[24] Erdelt K, Pinheiro Dias Engler ML, Beuer F, et al. Computable translucency as a function of thickness in a multi-layered zirconia. J Prosthet Dent. 2019;121(4):683–689. 30527568 10.1016/j.prosdent.2018.08.013

[25] Elsaka SE. Optical and mechanical properties of newly developed monolithic multilayer zirconia. J Prosthodont. 2019;28(1):e279–e284. 29239067 10.1111/jopr.12730

[26] Harada K, Raigrodski AJ, Chung KH, et al. A comparative evaluation of the translucency of zirconias and lithium disilicate for monolithic restorations. J Prosthet Dent. 2016;116(2):257–263. 26994676 10.1016/j.prosdent.2015.11.019

[27] Aden A, Anderson P, Burnett GR, et al. Longitudinal correlation of 3D OCT to detect early stage erosion in bovine enamel. Biomed Opt Express. 2017;8(2):954–973. 28270996 10.1364/BOE.8.000954 PMC5330568

